# How do economic and public finance statuses affect policy responses during a pandemic? – learning from the COVID-19 first wave

**DOI:** 10.1186/s12889-022-13209-6

**Published:** 2022-04-19

**Authors:** Tasadduq Imam, Shahadat Uddin

**Affiliations:** 1 School of Business and Law, CQUniversity (Melbourne Campus), Melbourne, VIC 3000 Australia; 2 School of Project Management, Faculty of Engineering, The University of Sydney, Forest Lodge, NSW 2037 Australia

**Keywords:** COVID-19, Health policies, Economic status, Pandemic, Public finance

## Abstract

**Background:**

In the time of a pandemic, it is typical for public health bodies to collaborate with epidemiologists to design health policies both at national and international levels for controlling the spread. A point largely overlooked in literature is the extent economic capability and public finance status can influence the policy responses of countries during a pandemic situation. This article fills this gap by considering 12 public health and 7 economic measures (i.e., policies) in 200 countries during the COVID-19 first wave, with countries grouped across income categories.

**Methods:**

We apply statistical analysis, inclusive of regression models, to assess the impact of economic capability and public finance status on policy responses. Multiple open-access datasets are used in this research, and information from the hybrid sources are cumulated as samples. In our analysis, we consider variables including population characteristics (population size, density) and economic and public finance status (GDR, current account balance, government surplus/deficit) further to policy responses across public health and economic measures. Additionally, we consider infection rates across countries and the institution of the measures relative to infection rate.

**Results:**

Results suggest that countries from all income groups have favoured public health measures like school closures and travel bans, and economic measures like influencing interest rates. However, strong economy countries have more adopted technological monitoring than low-income countries. Contrarily, low-income countries have preferred traditional measures like curfew and obligatory mask-wearing. GDP per capita was a statistically significant factor influencing the institution of both public health and economic measures. Government finance statuses like current account balance and surplus/deficit were also significant factors influencing economic measures.

**Conclusions:**

Overall, the research reveals that, further to biological characteristics, policymakers and epidemiologists can consider the economic and public finance contexts when suggesting health responses to a pandemic. This, in turn, calls for more international cooperation on economic terms further to public health terms.

**Supplementary Information:**

The online version contains supplementary material available at 10.1186/s12889-022-13209-6.

## Introduction

In the face of a pandemic, it is typical for international and national public health bodies, in collaboration with epidemiologists, to prescribe some standard actions and initiate some measures to protect public health. The COVID-19 pandemic was no exception, leading to governments of different countries instituting different public health policies as a response. However, some health emergency measures like lockdowns and other restrictions carry economic costs, and not all countries have similar abilities to absorb such costs. Further, financial capabilities of governments, characterised through government finance status [1], can largely influence their investment and spending, and subsequently their policy responses when confronting health emergencies like that experienced during the COVID-19 first wave. However, to the best of the authors’ knowledge, the economic and Government finance status of the Government in relation to policy responses, especially when the emergency was as unprecedented as the COVID-19 first wave, have not received much attention in the literature. This article, by exploring the extent economic and Government finance (also referred as public finance) statuses affected the public health and economic measures across 200 countries, provides insights in this regard. Such insights can be helpful to epidemiologists and policymakers, especially when suggesting public health policies during a pandemic situation and predicting the effectiveness of the strategies.

Notably, governments’ actions during infectious disease outbreaks have received some attention in the literature. A closely related pandemic before the COVID-19 is the H1N1 virus outbreak in 2009, and it was a flu-like disease [2]. While initially reported in Mexico and the US, the virus quickly spread across multiple countries before slowing down because of protective public health measures [3]. Like the current COVID-19 outbreak, the H1N1 2009 outbreak also presented uncertain situations and challenging decision making contexts for policymakers and officials [4]. In the post-epidemic, the actions of various governments have come under scrutiny. Freimuth et al. [5], for example, identified variations among the different US demographic groups concerning their trust of the Government’s advice in the early stage of the H1N1 outbreak. Chambers et al. [6] argued for involvements by local policymakers for effective control of epidemic by considering the progress of H1N1 and Governmental responses in the U.K. Waller et al. [7] explored Australia’s response to the H1N1 pandemic and reflected on the need of flexibility in protecting public health during such outbreak. Henry [8] assessed the lessons learned from the H1N1 spread in Canada and emphasised consistency in implementing public health actions and transparent communications. Similarly, Liang et al. [9] noted the Chinese Government’s response to the H1N1 outbreak and the impacts different measures had on the spread.

The current COVID-19 outbreak, despite a virus caused disease and initially predicted to be a flu-like ailment, is different in characteristics. The disease, in its first-wave, appeared to have a higher prevalence in older patients than those who experienced H1N1 [10]. Further, the high-risk group of H1N1 included pregnant women, while early research suggested COVID-19 as more common in men [6, 10]. Research also indicated that older individuals had less risk of getting H1N1 [11], while a study soon following the COVID-19 outbreak identified children as less susceptible to COVID-19 [12]. Indeed, biological differences exist between the COVID-19 and past flu virus strains [13].

Thus, notwithstanding some similarities of events and despite different nations having different plans to confront health emergencies, the COVID-19 pandemic with its distinctive characteristics came as an unprecedented event across nations. This leads to the interesting question: how do economic and Government finance statuses (i.e., public finance status) influence the governments’ policy responses when facing such unprecedented health emergencies?

There are ongoing studies on the pandemic from various perspectives. Research, for example, identifies quickness of governmental response, social characteristics, and public perception as contributing factors to the early success of containing COVID-19 in Denmark [14]. Imai [15], while considering the COVID-19 context in a Japanese hospital, emphasises trust between health workers and their organisations for job motivation and retention of health workers. Hale et al. [16] present a custom dataset and report some early outcomes on variation in governmental responses to COVID-19 onslaught. Fang et al. [17] argue for different government interventions to contain transmission of the virus. McKibbin and Fernando [18] highlight the pandemic’s macroeconomic costs, even when contained through restrictive measures. Hopman et al. [19] suggest the rapid implementation of infection control and prevention and media involvement to manage COVID-19 outbreaks in low-income and middle-income countries. Wang et al. [20] reflect on the management of COVID-19 in Taiwan due to timely public policy measures and transparent communication. Another research explores the positive impacts of early surveillance and contact tracking on the COVID-19 outbreak in Singapore [21]. Research suggests that developing countries can find containing pandemics more challenging than industrialised countries due to a lack of medical infrastructures and clinical interventions [22]. A recent report, which categorises 199 countries according to income group, identifies the difference in COVID-19 testing capability of the countries [23].

However, research exploring the linkage of economic and public finance statuses with the institution of measures during a pandemic is mostly lacking to our best knowledge. This research fills this gap. Since more pandemic waves may appear at different locations until discovering an effective vaccine for the different variants of the disease, assessing the linkage can provide valuable insights for future health policy measures and planning.

## Methods

Multiple open-access datasets are used in this research. First, the *Government’s’ Response to the COVID-19 Database* is used to extract daily infection and death information for the 200 countries considered in this study – we use and evaluate information from Jan 1, 2020, to Jun 30, 2020 [24]. This dataset also contains calendar information regarding when a country has implemented a mitigation measure and collates related information from multiple other sources [24–27].

Second, a dataset from the World Bank (WB) has been used to classify the income group of a country [28]. WB categorises all countries into four income groups. They are ‘*high*’, ‘*upper-middle*’, ‘*lower-middle*’ and ‘*low*’. We further use the *World Economic Outlook* dataset from the International Monetary Fund to collect information about government finance statistics and GDP per capita of the considered countries [29]. Finally, the *United Nations World Population Perspectives 2019* [30] dataset is used to collect the population size, population density, and age distribution of each country considered in this research.

Notably, the COVID-19 response dataset lists 228 countries [24]. However, for some countries, either information is missing, or the corresponding countries’ data are unavailable from the other datasets considered in this research. Hence, we consider explicitly those countries for which most of the needed information, including infection and death incidences and population characteristics, are available. This consideration leads to a selection of 200 countries. Out of these 200 countries, 75, 52, 43, and 30 countries belong, respectively, to the *‘high’*, *‘upper-middle’*, *‘lower-middle’*, and *‘low’* income groups.

We further augment the response dataset [24] by considering the infection rate determined on a population size of 10 million. Thus, if *P* is the population size of a country, and *I* is the number of infection cases, the infection rate is: $\frac{I}{\left(\frac{P}{10m}\right)}\times 100\%$.

The original dataset contains public health and economic measures (i.e., policies) implemented nationally and partially or locally. However, we focused on governments at a national level and considered that not all countries qualify for local or partial measures because of their population size or land area. Thus, our consideration leads to an unbiased comparison. We assess 12 public health and 7 economic measures which have a value of either 1 or 0 indicating, respectively, if the respective health policy was or was not in place on a specific date in a country. The consideration of these 19 measures also correspond to their usages in several recent COVID-19 studies (e.g. [31, 32],), and these can hence assist in gaining insights on Governments’ responses during the COVID-19 onslaught.

The 12 public health measures include [24]: *‘school’* (schools were closed), *‘domestic lockdown’* (there were lockdowns at a national level), *‘travel’* (a travel ban was imposed), *‘curfew’* (curfew was declared), *‘mass gathering’* (restrictions on mass gathering of individuals), *‘election’* (the election was postponed), *‘sport’* (sports and large events were restricted), *‘restaurant’* (restaurants and bars were shut down), *‘testing’* (there was a public testing policy), *‘surveillance’* (technologies like mobile app or bracelet were used for surveillance), *‘masks’* (public instructed to wear masks), and *‘state emergency’* (a state of emergency was declared).

The 7 economic measures include [24]: *‘cash’* (the Government considered cash transfers), *‘wage’* (there were some support measures covering wage), *‘credit scheme’* (the country instituted credit schemes), *‘tax credit’* (the Government adopted a fiscal policy like providing tax credits), *‘tax delays’* (there was a fiscal policy like delays on tax), *‘export’* (the country had measures supporting traders), and *‘interest rate’* (if a monetary policy like lowering the interest rates was adopted).

We use the R statistical software and its various packages [33–35] to conduct analysis and present the outcomes. The publicly available original dataset [24] contains information on the implementation of the 19 measures by country basis across dates, and we consider some derived information from the dataset in our research. We note the percentage of countries within each income group implementing each of the measures, as well as the differences in time of implementing the measures and the changes in the implementation of measures relative to changes in infection rate, to gain an insight of variations across countries and the influences respective economic statuses had on the implementations. We, further, test the significance of economic and public finance statuses, expressed in terms of GDP, current account balance, and government surpluses/deficits, further to population characteristics, on implementation of the measures by a set of regression models. Further details are presented in the next section.

## Results

Table 1 shows the implementation statistics of various measures by four different income groups of countries for the considered period of COVID-19 first wave: Jan 1, 2020, to Jun 30, 2020. Each row of the Table shows the count of countries from each income group that implemented the respective measures. Notably, some countries have adopted a measure on a date and then lifted it on another date. We have considered each enacting of measure as one count of implementation of the respective measure for consistent comparison. Further, each income group comprises a different number of countries. To facilitate unbiased comparison across groups, we consider the percentage of countries adopting a measure relative to the total number of countries within an income group. Additionally, the income group most adopting each measure is noted.

**Table 1:** The implementation statistics of various measures by different income-based country groups. The shaded cells represent the highest percentage value for the corresponding rows

Measure	Income-based country group			
	*High (75)*	*Upper middle (52)*	*Lower middle (43)*	*Low (30)*
*Public health measures*				
School	64 (85%)	51 (98%)	42 (98%)	29 (97%)
Domestic lockdown	43 (57%)	45 (87%)	31 (72%)	17 (57%)
Travel	55 (73%)	51 (98%)	42 (98%)	29 (97%)
Curfew	15 (20%)	36 (69%)	14 (33%)	19 (63%)
Mass gathering	52 (69%)	43 (83%)	40 (93%)	24 (80%)
Election	17 (23%)	17 (33%)	12 (28%)	5 (17%)
Sport	51 (68%)	36 (69%)	30 (70%)	23 (77%)
Restaurant	49 (65%)	39 (75%)	32 (74%)	20 (67%)
Testing	45 (60%)	25 (48%)	21 (49%)	11 (37%)
Masks	36 (48%)	36 (69%)	32 (74%)	17 (57%)
Surveillance	25 (33%)	8 (15%)	9 (21%)	1 (3%)
State emergency	38 (51%)	37 (71%)	24 (56%)	17 (57%)
*Economic measures*				
Cash	33 (44%)	38 (73%)	29 (67%)	13 (43%)
Wage	46 (61%)	33 (63%)	19 (44%)	8 (27%)
Credit scheme	43 (57%)	31 (60%)	20 (47%)	7 (23%)
Tax credit	34 (45%)	24 (46%)	28 (65%)	15 (50%)
Tax delay	43 (57%)	30 (58%)	27 (63%)	12 (40%)
Export	23 (31%)	17 (33%)	9 (21%)	5 (17%)
Interest rate	47 (63%)	32 (62%)	34 (79%)	22 (73%)

The results reveal that the public health measures of ‘*testing*’ (60%) and ‘*surveillance*’ (33%) have been implemented most times by the ‘*high*’ income countries. Low-income countries have favoured mostly the ‘sport’ measure (77%). The *‘upper-middle’* and *‘lower-middle’* income countries have been most active in instituting most public health measures, with the *‘upper-middle’* group topping the implementation across 7 of these measures. Concerning the economic measures, again, the *‘upper-middle’* and *‘lower-middle’* income countries have been most active, closely followed by the *‘high’* income countries for most measures. Noticeably, the *‘low’* income group has been less active in implementing economic measures than other income groups, with less than 25% of countries in the group instituting the *‘credit scheme’* and *‘export’* measures.

If Table 1 is assessed column-wise, all income groups’ top public health interventions appear to be the *‘school’* and *‘travel’* measures. The *‘low’* income group has further emphasized on *‘mass gathering’* (80%), *‘restaurant’* (67%), *‘curfew’* (63%), *‘domestic lockdown’* (57%), *‘masks’* (57%) and *‘state emergency’* (57%) measures. However, only one low-income country has implemented the *‘surveillance’* measure (3%). Indeed, *‘surveillance’* measure implementation is lower than 25% for the *upper-middle’* and *‘lower-middle’* income countries. *‘High’* income countries, while having an emphasis on *‘mass gathering’* (69%), *‘sport’* (68%), *‘restaurant’* (65%), and *‘domestic lockdown’* (57%) further to other measures discussed, interestingly had the least implementation of *‘curfew’* (20%) and *‘masks’* (48%) measures.

Concerning economic measures, the top interventions favoured by the *‘high’* income group are the *‘interest rate’* (63%) and *‘wage’* (61%) measures. The *‘interest rate’* measure is a widely implemented intervention also by the *‘lower-middle’* and *‘low’* income countries, whereas *‘upper-middle’* income countries have primarily opted for the *‘cash’* measure (73%). Overall, Table 2 indicates differences in implementing measures across countries, especially economic measures, due to their economic status.

**Table 2:** Regression models relating the average count of measures to population and economic and public finance characteristics

	Average total measures		Average total public health measures		Average total economic measures	
	*Estimates*	*Standard Error*	*Estimates*	*Standard Error*	*Estimates*	*Standard Error*
(Intercept)	1.0162	1.9383	1.7952	1.3977	−0.7790	1.0730
log(*PopSize*_*i*_)	**0.6423** ^***^	0.0917	**0.3485** ^***^	0.0661	**0.2938** ^***^	0.0507
log (*PopDensity*_*i*_)	−0.0417	0.1214	−0.0832	0.0875	0.0415	0.0672
*Perc*0*to*19_*i*_	−0.0220	0.0157	−0.0141	0.0113	−0.0080	0.0087
*Perc*20*to*64_*i*_	−0.0160	0.0194	−0.0147	0.0140	−0.0013	0.0107
log (*GDP*_*i*_)	**0.9497** ^***^	0.2180	**0.4656** ^**^	0.1572	**0.4841** ^***^	0.1207
*CABalance*_*i*_	**−0.0214** ^*^	0.0102	−0.0086	0.0073	**−0.0128** ^*^	0.0056
*GovtSurplus*_*i*_	0.0074	0.0122	−0.0074	0.0088	**0.0148** ^*^	0.0067
N	174		174		174	
Adjusted R^2^	40.6%		24.1%		37.4%	

^*^p < 0.05

^**^p < 0.01

^***^p < 0.001

**Fig. 1:**
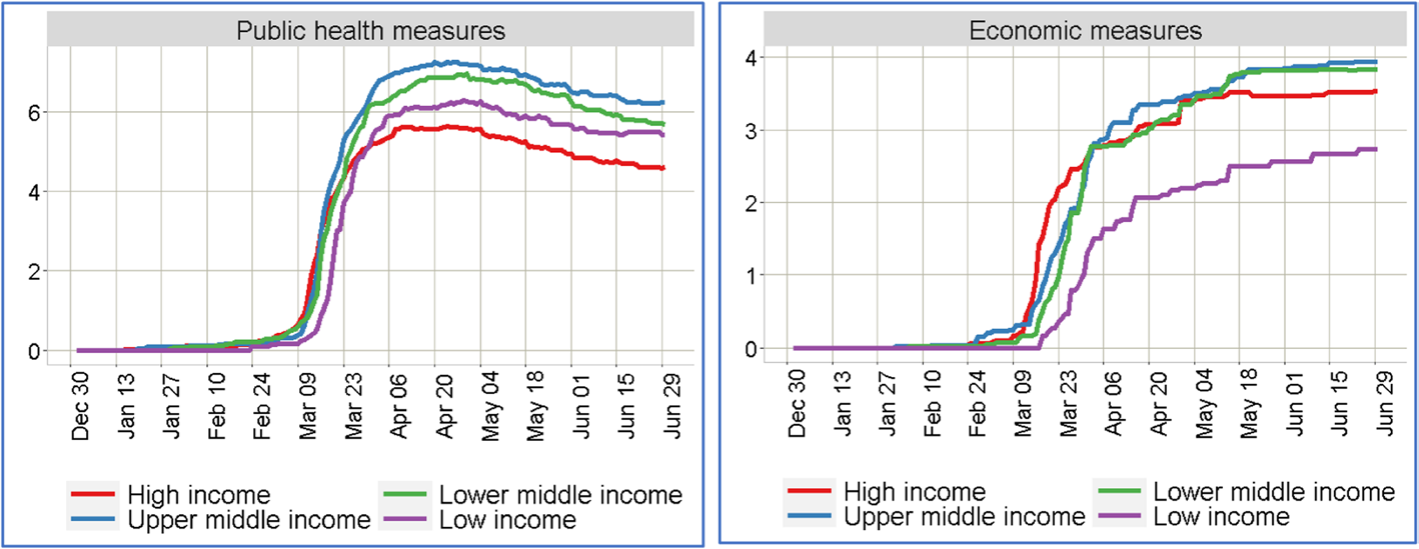
The average number of implemented measures over time by four different income group nations

Figure 1 illustrates the average number of implemented public health and economic measures by the governments of different income-based country groups. As revealed, for all income groups, the implementation rates for the public health measures considered in this research exhibit a sharp increase between Mar 10, 2020, and Mar 24, 2020. After reaching an implementation rate of around five to six, these sharp increases have flattened for all groups, and there is a decrease in average measures, especially following mid-May. This is an indication that several countries assumed the first wave of the virus outbreak was over in May and thereby gradually lifted some public health restrictions. On the other hand, for the economic measures, a similar sharp increase is noted between Mar 10, 2020, and Mar 31, 2020. Interestingly, the implementation rate of economic measures gradually increased over time – an indication that, while countries eased the public health restrictions at the downside of the first wave of COVID-19 outbreak, the respective governments shifted focus to economic consequences and instituted relevant measures accordingly.

We also conceptualise the change in the average number of total public health and economic measures with the change in the average infection rate for each income group. In calculating so, we first consider some thresholds of infection rates. We determine the total public health and economic measures that each country instituted before its cumulative infection rate surpassed a threshold. Let *t* be a threshold infection rate; *n* be the number of countries within a particular income group; and *x*_1_, *x*_2_, …, *x*_*n*_ be the total measures (public health measures or economic measures) instituted, respectively, by the countries within the group before their respective cumulative infection rate ,*I*_*i*_ : *i* = 1…*n*, crosses the threshold *t*. The average infection rate ($\frac{\sum_{i=1}^n{I}_i}{n}$), the average number of instituted measures ($\frac{\sum_{i=1}^n{x}_i}{n}$), and the standard deviation of the number of instituted measures are then determined for each income group for the respective threshold.

**Fig. 2:**
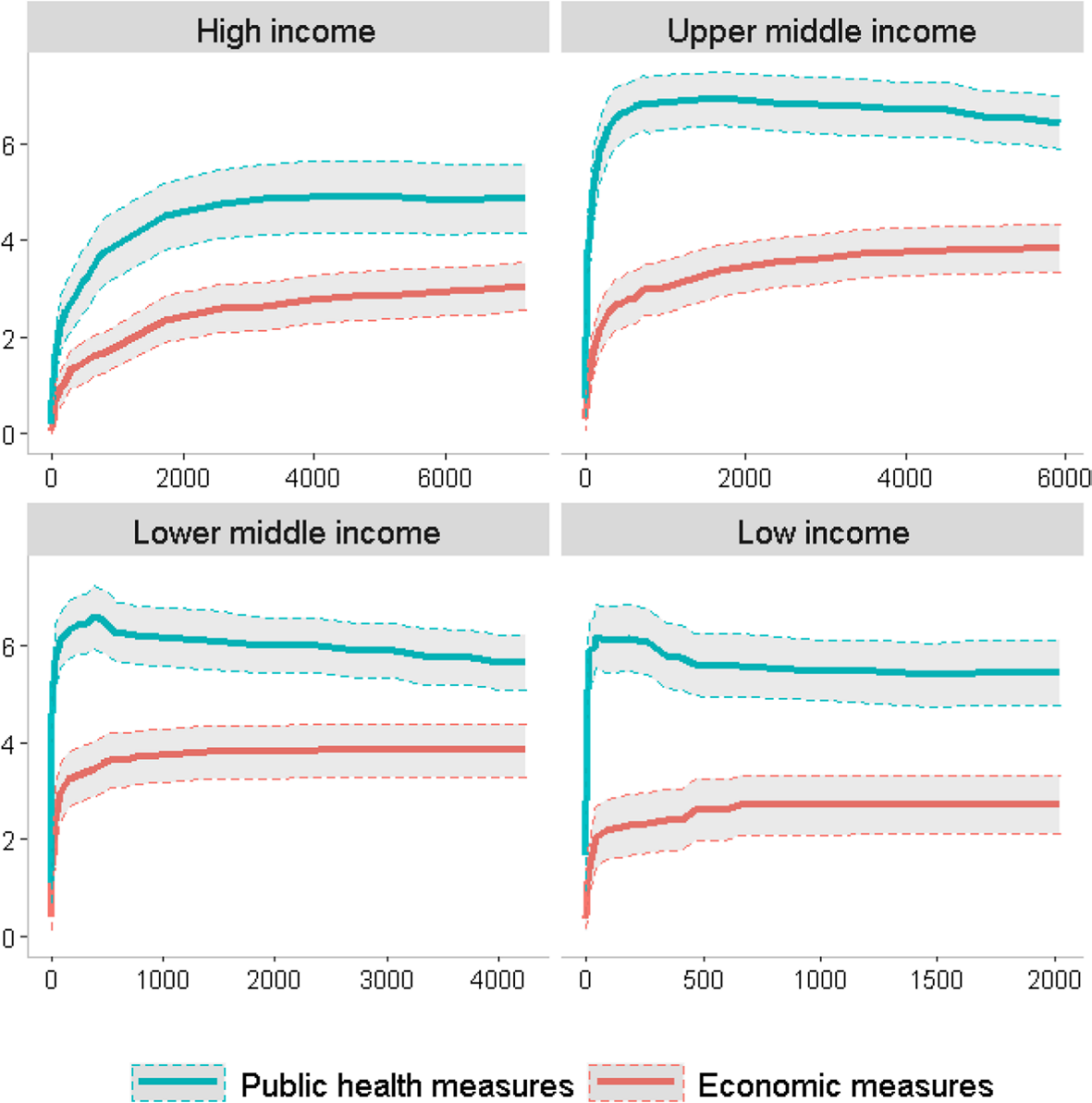
Changes in the average number of total public health and economic measures by different income group nations relative to the average cumulative infection rate changes for the considered period. The dotted lines and greyed area represent the 95% confidence interval for the respective averages

Figure 2 subsequently presents the average number of respective measures with a 95% confidence interval against the changes in average infection rate for different thresholds. As notable, the average number of public health measures has exceeded the average number of economic measures for each income group. Further, for the high income and upper-middle-income countries, there has been a gradual increase in economic measures with increased infection rates. By contrast, both lower-middle- and low-income countries have remained steady concerning the institution of economic measures relative to infection rates. Another notable aspect is that low-income countries have generally instituted fewer economic measures than higher-income groups. This is another indication that the economic conditions of countries had an impact on their institution of measures.

To gain further insights, we hypothesise a government’s institution of measures has been influenced by the respective country’s population size, density, and age distribution during the COVID-19 first wave and consider the following regression model:

1 $${Y}_i={\upbeta}_0+{\upbeta}_1\ \log \left({PopSize}_i\right)+{\upbeta}_2\log \left({PopDensity}_i\right)+{\upbeta}_3\ Perc0 to{19}_i+{\upbeta}_4\ \mathrm{Perc}20\mathrm{to}{64}_i+\kern2.25em {\upbeta}_5\ \log \left({GDP}_i\right)+{\upbeta}_6\ CABalanc{e}_i+{\upbeta}_7\ GovtSurplu{s}_i$$

where,

*Y*_*i*_: average number of measures adopted by country *i* (average over the considered period).

*PopSize*_*i*_: population size (millions).

*PopDensity*_*i*_: population density (persons per km^2^).

*Perc*0*to*19_*i*_: percentage of the total population aged 0-19 (%).

*Perc*20*to*64_*i*_: percentage of the total population aged 20-64 (%).

*GDP*_*i*_: per capita GDP in 2019 ($ per person).

*CABalance*_*i*_: Average of recent 5 years’ current account balance expressed as a percentage of GDP (%).

*GovtSurplus*_*i*_: Average of recent 5 years’ Government’s surplus or deficit expressed as a percentage of GDP (%).

There may be a question raised concerning the representation of the 19 measures, as explained in Section 2, in terms of a single dependent variable: *Y* (i.e., the average number of responses enacted by a country), as in Eq. (1). We conceptualise that enacting different types of measures by a country’s Government, from a summative perspective, also reflects its ability in managing a pandemic situation by such measures, and which is an important aspect especially with research noting not all countries could maintain or implement some hard measures like lockdowns, curfews, and social isolations due to economic and population contexts [36–38]. Thus, it is interesting to explore the extent the countries’ population context like population size, density, and age distribution and economic and public finance statuses affected the respective Government’s adoption of measures – the rationale behind the hypothesis in Eq. (1).

Indeed, the hypothesis is motivated by past epidemiological events and information available to governments during the early outbreak of COVID-19. Quarantine, self-isolation, and lockdowns measures have historically been used in controlling epidemics, mainly to avoid the person-to-person spread of the disease. It can be hypothesised that a country with large population size or large population density is likely to behave differently in instituting such measures to control an epidemic from countries with a smaller population or lower population density.

Further, as evident from Table 1, the *‘school’* measure has achieved significant attention across income groups during the current pandemic. Potentially, this is a consequence of the last similar pandemic (H1N1) that was fatal to children. Also, for most countries, the age group 20-64 constitutes the working-age individuals who are more likely to go outside the home for work and thereby are at high risk of being exposed to the disease during an epidemic. Thus, it can be hypothesised that the populace’s age distribution can also influence the institution of epidemic prevention measures within a pandemic situation. Notably, considering the percentage of the population in the age groups 0-19 and 20-64 also implicitly captures the influence of the age group at 65+, the 65+ group is not explicitly considered in the regression model to avoid collinearities.

In Eq. (1), we also consider the countries’ economic and public financial statuses. The GDP per capita relates to the income level of a country and, as evident from previous analysis, is likely to have influenced the policy responses. We hypothesise that governments of countries with higher GDP per capita will behave differently from the Government of the countries with lower GDP due to economic costs associated with the institution of disease protection measures. Further, such GDP variations may also reflect the respective governments’ economic orientations and subsequent public policy actions. Thus, we consider the annual GDP of countries in the year just before the pandemic as an influential factor.

Besides, with COVID-19 being a global pandemic, it makes sense to consider the government finance status like the national current account balance and Government’s surplus and deficit. The current account balance primarily reflects the gap between a country’s export and import of goods and services, with a negative balance indicating more imports than exports. If an economy mainly relies on imports or exports, it is typical for a government to consider this before instituting a restrictive measure like a lockdown. The government surplus/deficit suggests the difference between a government’s revenues and spending. We hypothesise that the recent average of these government finance measures reflects the respective governments’ financial capability, which subsequently has influenced their health policy planning and the implementation of measures.

We assess the stated hypotheses by considering the average count of total measures (public health measures + economic measures), public health measures, and economic measures, respectively, as dependent variables in Eq. (1) and noting the significance of coefficients for the three underlying models. In such an assessment, we focus on Jan 1, 2020, to Apr 30, 2020. As evident from Fig. 1 and discussed earlier, several countries lifted some restrictive public health measures from early May onwards, indicating a likely end of the pandemic’s first wave for several countries. Hence, we concentrate on the period before May when the pandemic was in the peak stage. Also, among the 200 countries, information on population characteristics or economic conditions are unavailable for a few countries from the respective data sources. Due to its inherent statistical nature, the regression model considers only those countries for which all information modelled by Eq. (1) is available. Additional file 1 shows the countries considered by the regression model along with the respective values for dependent variables.

Table 2 presents the outcomes of the regression analysis. It is notable that population size and GDP consistently appear as significant variables for all three models. This implies, with increasing population size and increased GDP, countries have instituted more public health and economic measures. Further, the current account balance appears a significant predictor for both total and economic measures, while government surplus/deficit also emerges as a significant factor for economic measures. Interestingly, other population measures like population density and age distribution do not appear significant for any models. Overall, the regression model reinforces the influence of countries’ economic and public finance status in their policy responses during the COVID-19 first wave.

## Discussion and implications

Overall, our analysis reveals noticeable differences among countries concerning their responses during the COVID-19 first wave. An interesting aspect is the broader use of technological monitoring like surveillance among high-income countries than lower-income countries. As noted by Mohanty et al. [39], with early detection playing a principal role in containing and managing an epidemic, technologies like mobile apps can collate data from multiple sources and communicate useful information. Further, as Tréguer [40] highlights, digital surveillance allows monitoring individual actions and contact tracing at a massive scale with a less cost and ease of policing the measures. Another recent research shows mobile apps and surveillance’s effectiveness in containing the spread of COVID-19 [41]. Arguably, high-income countries have favoured exploiting technologies for managing the COVID-19 onslaughts and which reflect their high technical capability. By contrast, low-income countries appear to have focused on traditional quarantine and isolation measures like restricting travel bans, restaurants, mass gatherings, and sports. While other income group countries have also considered these traditional measures, the general absence of surveillance measures in the low-income group is noticeable. Noticeably, the “Global Innovation Index 2019” lists high and upper-middle-income countries as topping the ranks, while several low-income countries rank low [42]. The lower use of technological measures during COVID-19 in low-income countries reflects this difference in their technological capability and innovation orientation compared to the higher income group countries.

This discrepancy can also be conceptualised from the difference in internet infrastructure and technological contexts between the high- and low-income counties. Research notes low income countries are often unable to adopt advanced health systems like surveillance of injuries due to affordability and lack of internet infrastructure [43]. Research also points to inconsistency in internet connection as barriers to the spread of telepathology services in low and middle-income countries [44]. Potentially, similar issues concerning internet infrastructure, affordability, and connection reliability may have also played a role in the lower use of technological monitoring during the COVID-19 early wave in the low-income countries.

The lower implementation of masks in high-income countries is worth noting. Research before the COVID-19 pandemic has argued the effectiveness of masks in controlling the spread of flu-like diseases [45]. There have also been concerns in the early stage of the COVID-19 pandemic that wearing masks may provide the wearer with a false sense of security rather than preventing the disease [46]. Indeed, research notes the reluctance by public health bodies to prescribe masks during the early wave of COVID-19, especially with uncertainties about symptoms and spread characteristics of the disease [36]. Feeling of discomfort and social isolations have also been noted as case of mask resistance in some Western countries [36]. Further, while many rich countries during the COVID-19 had the choice concerning implementation of lockdowns, poorer countries often were forced to lift such bans early to keep the economy open [47]. Also, compared to some Western countries some Asian countries were early adopters of masks due to their past experience of using masks to contain pandemics [47]. Arguably, these uncertainties and situational contexts explain the lower acceptance of masks in some high-income countries compared to low-income countries.

The adoption of economic measures also shows notable differences. With a relatively higher economic strength, the high and upper-middle-income group countries were more frequent to support wages than the lower-middle- and low-income group countries. The high and upper- and lower-middle-income countries were also more frequent in initiating cash, credit, and export supports. By contrast, most low-income countries favoured the lowering of interest rates as an economic measure. Thus, while the countries with economic strength implemented direct intervention like cash and trade measures to absorb the economic effect of public health policies and support their businesses and workers, many low-income countries relied mainly on indirect economic intervention like lowering the interest rate for boosting and sustaining economic growth.

The regression model further characterises the impact of economic status and government financial status on public health measures during a pandemic. GDP has had a notable positive influence on the average number of measures adopted by countries in response to the COVID-19 onslaught. A significant positive effect of GDP is also noticed for the average count of public health and economic measures. Countries also had enacted more measures with increasing population size, potentially indicating that the respective governments considered the mass scale detrimental impact the virus can have on their population.

What is interesting, however, and which has not been well explored in the relevant literature, is the noticeable impact of government financial statuses on the institution of measures. The current account balance appears as a significant factor in the institution of total measures and economic measures. A negative current account balance implies a country has more imports than exports. Thus, a negative coefficient associated with the current account balance implies that having more imports led governments to institute more economic measures, potentially to absorb the cost of restrictive public health measures. The significant positive influence of government surplus/deficit on economic measures also makes sense. This implies that governments with higher financial capabilities have instituted more economic measures than governments with lower financial capabilities. Interestingly, population characteristics like population density and age distribution do not appear as significant factors.

Overall, when facing a pandemic, different countries’ governments face both public health and economic challenges. During such crises, international and national health bodies, in collaboration with epidemiologists, often advise specific health directions. However, as the COVID-19 responses reveal, economic and financial statuses of the countries can either limit or facilitate the government policy responses, placing some countries, especially those from the higher income group, at an advantage while other countries, especially those from the low-income group, at a disadvantaged position.

A public health emergency like a pandemic, however, can often spread quickly across regions within the contemporary highly connected society and controls just at a national level may not be fruitful. Simultaneously, a sole focus on public health but not on the economic context may not result in effective prevention, especially if the respective Government and the populace is unable to cope with the economic pressure that comes with instituting the public health measures.

Thus, when a global pandemic arises, epidemiologists and policymakers across countries can play a role that go beyond providing medical advice and suggesting policies. They also need to consider the costs of such actions relative to the economic context and governmental financial status, especially since the actions may not be sustainable otherwise. A greater inter-collaboration between countries can further be effective in this respect. Higher-income countries and global bodies, for instance, can provide financial support to countries with weaker economies or lower financial capability to absorb restrictive public health policies’ economic costs. On the flip side, public health entities in lower-income countries can plan beyond designing health policies and procedures for epidemics. They may engage at various political and international levels to ensure sufficient funding to cushion for the restrictive policies’ economic costs or to keep the economy moving within epidemiological restrictions.

In this respect, it is worth referring to the pandemic bonds issued by the World Bank which aimed at raising emergency funds while rewarding investors for the risk [48, 49]. Even though the effectiveness of the scheme has come under debate [48, 49], the analysis in this research arguably calls for the establishment of similar and broader level financial instruments, potentially funded by entities at the international level, towards reducing the divide in actions between countries when facing a pandemic. This, in turn, may lead to better addressing of a global health emergency – a point for further research and debate.

## Conclusion

In summary, an assessment of government responses for 200 countries in the early stages of the COVID-19 outbreak shows noticeable variations. The research implies that not all countries can similarly react to pandemic outbreaks, and the economic constraints and the Government’s financial status play a significant role in their capability to institute public health responses. The difference becomes even more contrasting when such a public health emergency, as the first wave of the COVID-19, is unprecedented and unique.

Like any other study, this research also has some limitations. First, we did not consider the impact of any factor other than that covered by the original dataset, and which may influence the 19 public health and economic measures considered in this study. For example, there are country level resources, like number of medical services, availability of specialists, connections between medical service providers, and economic support behind health services, that can affect the spread and control of pandemics. There are also population level characteristics, like the traditional practice of populace in seeking medical services and spread characteristics of the infection relative to demographic attributes, which can characterise pandemic within a community. Thus, while these factors are not considered due to unavailability of such information in the original dataset, a future work will explore these additional factors as an extension. Also, this study has not considered the precedencies between multiple measures in developing the regression models. For example, ‘*domestic lockdown*’ generally precedes the ‘*state lockdown*’ for a country while attempting to take steps to control the spread of COVID-19. A future work will explore this sequence of policy implementations with respect to the economic status of countries.

Overall, despite a few limitations, this research provides insights on the extent economic and public finance statuses of a country can impact policy measures for an uncertain pandemic situation. Thus, while it is typical during a pandemic situation for health agencies at country and global levels to prescribe some common standards, the differences in economic and Government financial status (i.e., public financial status) across countries cannot be ignored. With this in consideration, there is a need for greater national and international collaboration among policymakers and epidemiologists on economic terms further to public health terms in policy design and planning against a future global pandemic – a point often overlooked yet which needs further research and political reflections.

## Supplementary Information

**Additional file 1.** Countries considered in the regression model and the respective dependent variables

## Data Availability

Data used for this research are publicly available from the openICPSR, the World Bank, the International Monetary Fund, and the United Nations websites [24, 28–30].
