# Supplementary material for: How do economic and public finance statuses affect policy responses during a pandemic? – learning from the COVID-19 first wave
Source: BMC Public Health. 2022 Apr 19;22:785. doi: 10.1186/s12889-022-13209-6 (PMC9016378; doi:10.1186/s12889-022-13209-6)
Supplement: Supplementary file 1 — Additional file 1. Countries considered in the regression model and the respective dependent variables [file 12889_2022_13209_MOESM1_ESM.docx]

**Additional file 1 Countries considered in the regression model and the respective dependent variables**

| **Country** | **Avg. Public Health Measures** | **Avg. Economic Measures** | **Avg. Total Measures** |
| --- | --- | --- | --- |
| Afghanistan | 2.38 | 0.35 | 2.73 |
| Albania | 3.02 | 1.23 | 4.25 |
| Algeria | 1.95 | 0.96 | 2.91 |
| Angola | 1.78 | 0.69 | 2.46 |
| Antigua and Barbuda | 1.73 | 0.89 | 2.62 |
| Argentina | 3.23 | 0.32 | 3.55 |
| Armenia | 2.65 | 0.79 | 3.45 |
| Aruba | 0.38 | 0.60 | 0.98 |
| Australia | 3.05 | 2.43 | 5.48 |
| Austria | 3.26 | 2.00 | 5.26 |
| Azerbaijan | 2.28 | 0.88 | 3.16 |
| Bahamas | 2.07 | 1.10 | 3.17 |
| Bahrain | 2.99 | 1.86 | 4.85 |
| Bangladesh | 1.56 | 1.34 | 2.90 |
| Barbados | 2.74 | 1.27 | 4.01 |
| Belarus | 1.02 | 0.48 | 1.50 |
| Belgium | 2.60 | 0.98 | 3.58 |
| Belize | 1.31 | 0.74 | 2.06 |
| Benin | 2.45 | 0.23 | 2.68 |
| Bhutan | 1.60 | 0.14 | 1.74 |
| Bolivia | 3.33 | 0.02 | 3.36 |
| Bosnia and Herzegovina | 3.93 | 0.88 | 4.81 |
| Botswana | 2.69 | 0.77 | 3.45 |
| Brazil | 2.09 | 1.52 | 3.61 |
| Brunei Darussalam | 1.82 | 0.50 | 2.31 |
| Bulgaria | 3.13 | 0.94 | 4.07 |
| Burkina Faso | 2.61 | 0.82 | 3.43 |
| Burundi | 0.34 | 0.87 | 1.21 |
| Cabo Verde | 1.58 | 1.64 | 3.21 |
| Cambodia | 1.70 | 0.79 | 2.50 |
| Cameroon | 2.31 | 0.31 | 2.62 |
| Canada | 3.03 | 2.11 | 5.14 |
| Central African Republic | 1.58 | 0.29 | 1.87 |
| Chad | 2.62 | 0.29 | 2.91 |
| Chile | 3.48 | 1.26 | 4.74 |
| China | 4.40 | 2.32 | 6.72 |
| Colombia | 2.54 | 1.97 | 4.50 |
| Comoros | 1.57 | 0.53 | 2.10 |
| Democratic Republic of the Congo | 2.72 | 0.67 | 3.39 |
| Costa Rica | 2.18 | 0.00 | 2.18 |
| Cote d'Ivoire | 2.88 | 1.53 | 4.40 |
| Croatia | 2.17 | 0.87 | 3.03 |
| Cyprus | 3.60 | 0.50 | 4.10 |
| Czechia | 3.23 | 1.16 | 4.39 |
| Congo | 1.81 | 0.72 | 2.53 |
| Denmark | 2.53 | 1.52 | 4.05 |
| Djibouti | 1.69 | 0.00 | 1.69 |
| Dominican Republic | 3.64 | 0.65 | 4.29 |
| Ecuador | 3.12 | 1.07 | 4.18 |
| Egypt | 1.82 | 1.79 | 3.60 |
| El Salvador | 2.36 | 0.00 | 2.36 |
| Equatorial Guinea | 2.28 | 0.61 | 2.89 |
| Eritrea | 1.46 | 0.00 | 1.46 |
| Estonia | 2.16 | 0.66 | 2.82 |
| Eswatini | 2.26 | 0.25 | 2.50 |
| Ethiopia | 2.19 | 1.22 | 3.41 |
| Fiji | 2.59 | 1.37 | 3.96 |
| Finland | 1.76 | 1.29 | 3.05 |
| France | 3.77 | 1.63 | 5.40 |
| Gabon | 3.39 | 0.75 | 4.14 |
| Gambia | 2.11 | 0.75 | 2.86 |
| Georgia | 2.94 | 0.82 | 3.76 |
| Germany | 3.12 | 1.88 | 5.00 |
| Ghana | 2.40 | 0.36 | 2.75 |
| Greece | 3.14 | 1.32 | 4.46 |
| Grenada | 2.36 | 1.39 | 3.74 |
| Guatemala | 2.65 | 1.69 | 4.34 |
| Guinea | 2.23 | 0.74 | 2.98 |
| Guinea-Bissau | 2.19 | 0.00 | 2.19 |
| Guyana | 2.20 | 1.03 | 3.23 |
| Haiti | 2.35 | 0.00 | 2.35 |
| Honduras | 2.37 | 1.16 | 3.53 |
| Hungary | 2.78 | 1.17 | 3.94 |
| Iceland | 2.92 | 1.45 | 4.36 |
| India | 3.26 | 0.90 | 4.16 |
| Indonesia | 3.06 | 1.36 | 4.41 |
| Iran | 1.21 | 0.61 | 1.82 |
| Iraq | 2.34 | 0.23 | 2.57 |
| Ireland | 2.80 | 2.03 | 4.83 |
| Israel | 2.94 | 2.12 | 5.06 |
| Italy | 4.74 | 1.89 | 6.63 |
| Jamaica | 1.32 | 0.45 | 1.77 |
| Japan | 3.10 | 1.52 | 4.62 |
| Jordan | 2.54 | 1.18 | 3.72 |
| Kazakhstan | 2.03 | 0.40 | 2.43 |
| Kenya | 2.74 | 0.93 | 3.67 |
| Kuwait | 3.47 | 0.48 | 3.95 |
| Kyrgyzstan | 2.70 | 0.74 | 3.45 |
| Laos | 2.31 | 1.31 | 3.61 |
| Latvia | 2.56 | 1.43 | 3.99 |
| Lebanon | 3.07 | 1.32 | 4.40 |
| Lesotho | 1.55 | 0.62 | 2.17 |
| Liberia | 2.26 | 0.00 | 2.26 |
| Libya | 3.42 | 0.00 | 3.42 |
| Lithuania | 3.43 | 1.53 | 4.96 |
| Luxembourg | 2.78 | 1.87 | 4.64 |
| Madagascar | 3.01 | 0.80 | 3.81 |
| Malawi | 1.98 | 0.98 | 2.96 |
| Malaysia | 2.33 | 2.65 | 4.98 |
| Maldives | 2.02 | 1.04 | 3.06 |
| Mali | 2.07 | 0.79 | 2.86 |
| Malta | 2.92 | 1.07 | 3.99 |
| Mauritania | 2.19 | 1.83 | 4.02 |
| Mauritius | 2.50 | 1.49 | 3.99 |
| Mexico | 2.49 | 1.29 | 3.78 |
| Moldova | 1.70 | 1.57 | 3.27 |
| Mongolia | 3.70 | 2.16 | 5.86 |
| Montenegro | 2.30 | 1.23 | 3.53 |
| Morocco | 3.25 | 1.53 | 4.78 |
| Mozambique | 2.17 | 0.16 | 2.32 |
| Myanmar | 1.88 | 1.54 | 3.42 |
| Namibia | 2.79 | 0.63 | 3.42 |
| Nepal | 1.67 | 0.80 | 2.47 |
| Netherlands | 1.91 | 1.38 | 3.29 |
| New Zealand | 2.45 | 2.09 | 4.55 |
| Nicaragua | 0.36 | 0.50 | 0.87 |
| Niger | 2.48 | 0.87 | 3.35 |
| Nigeria | 2.30 | 0.41 | 2.71 |
| North Macedonia | 2.01 | 0.66 | 2.67 |
| Norway | 2.27 | 1.39 | 3.66 |
| Oman | 1.75 | 1.43 | 3.18 |
| Pakistan | 3.37 | 1.88 | 5.26 |
| Panama | 2.83 | 0.38 | 3.21 |
| Papua New Guinea | 2.48 | 0.49 | 2.97 |
| Paraguay | 3.99 | 1.47 | 5.46 |
| Peru | 2.90 | 1.41 | 4.31 |
| Philippines | 4.01 | 1.23 | 5.24 |
| Poland | 3.21 | 0.95 | 4.17 |
| Portugal | 3.11 | 1.40 | 4.50 |
| Qatar | 2.20 | 0.38 | 2.58 |
| Republic of Korea | 2.56 | 1.07 | 3.63 |
| Romania | 3.17 | 2.03 | 5.21 |
| Russia | 2.36 | 1.64 | 4.01 |
| Rwanda | 2.07 | 1.10 | 3.17 |
| Saint Lucia | 2.55 | 0.81 | 3.36 |
| Saint Vincent and the Grenadines | 0.32 | 0.00 | 0.32 |
| São Tomé and Príncipe | 1.82 | 0.96 | 2.78 |
| Saudi Arabia | 2.83 | 1.44 | 4.27 |
| Senegal | 1.78 | 0.48 | 2.26 |
| Serbia | 2.89 | 1.58 | 4.47 |
| Seychelles | 2.38 | 0.65 | 3.03 |
| Sierra Leone | 1.74 | 0.73 | 2.47 |
| Singapore | 2.91 | 2.12 | 5.03 |
| Slovakia | 2.79 | 0.94 | 3.73 |
| Slovenia | 2.64 | 0.97 | 3.60 |
| South Africa | 3.23 | 1.71 | 4.94 |
| South Sudan | 1.49 | 0.06 | 1.55 |
| Spain | 3.21 | 2.44 | 5.64 |
| Sri Lanka | 2.31 | 1.29 | 3.60 |
| Sudan | 1.57 | 0.13 | 1.70 |
| Suriname | 2.10 | 0.19 | 2.29 |
| Sweden | 2.36 | 1.60 | 3.97 |
| Switzerland | 2.71 | 1.56 | 4.27 |
| Taiwan | 0.83 | 1.09 | 1.92 |
| Tajikistan | 0.79 | 0.03 | 0.83 |
| Tanzania | 1.21 | 0.00 | 1.21 |
| Thailand | 3.77 | 2.36 | 6.12 |
| Timor-Leste | 1.80 | 0.27 | 2.07 |
| Togo | 2.40 | 0.99 | 3.40 |
| Trinidad and Tobago | 2.53 | 1.98 | 4.51 |
| Tunisia | 3.67 | 1.71 | 5.38 |
| Turkey | 2.46 | 1.71 | 4.17 |
| Uganda | 2.06 | 1.42 | 3.48 |
| Ukraine | 2.64 | 1.61 | 4.26 |
| United Arab Emirates | 2.47 | 1.50 | 3.98 |
| United Kingdom | 3.40 | 2.16 | 5.56 |
| United States of America | 3.83 | 2.10 | 5.93 |
| Uruguay | 2.28 | 1.62 | 3.90 |
| Uzbekistan | 2.73 | 1.55 | 4.28 |
| Venezuela | 2.59 | 0.00 | 2.59 |
| Vietnam | 3.17 | 0.77 | 3.94 |
| Yemen | 2.13 | 0.00 | 2.13 |
| Zambia | 1.97 | 0.58 | 2.55 |
| Zimbabwe | 2.62 | 0.26 | 2.88 |
